# Enhanced oxygen transfer rate and bioprocess yield by using magnetite nanoparticles in fermentation media of erythromycin

**DOI:** 10.1186/s40199-014-0066-5

**Published:** 2014-09-16

**Authors:** Ghazal Labbeiki, Hossein Attar, Amir Heydarinasab, Sayed Sorkhabadi, Alimorad Rashidi

**Affiliations:** Department of Chemical Engineering, Science and Research Branch, Islamic Azad University(IAU), Tehran, Iran; Department of Pharmacology, School of Advanced Sciences and Technologies in Medicine, Tehran University of Medical Sciences, Tehran, Iran; Department of Toxicology and Pharmacology, Islamic Azad University of Pharmaceutical Sciences Branch, Tehran, Iran; Catalyst and Nanotechnology Division, Research Institute of Petroleum Industry, Tehran, Iran

**Keywords:** Bioprocess, Oxygen transfer rate, Mass transfer coefficient (KLa), Magnetite nanoparticles, Pharmaceutical biotechnology, Fermentation, Erythromycin, *Saccharopolyspora erythraea*

## Abstract

**Background:**

Magnetite nanoparticles have widespread biomedical applications. In the aerobic bioprocesses, oxygen is a limiting factor for the microbial metabolic rate; hence a high availability of oxygen in the medium is crucial for high fermentation productivity. This study aimed to examine the effect of using magnetite nanoparticles on oxygen transfer rate in erythromycin fermentation culture.

**Methods:**

Magnetite nanoparticles were synthetized through co-precipitation method. After observing the enhanced oxygen transfer rate in deionized water enriched with magnetite nanoparticles, these nanoparticles were used in the media of by *Saccharopolyspora erythraea* growth to explore their impact on erythromycin fermentation titer. Treatments comprised different concentrations of magnetite nanoparticles, (0, 0.005, 0.02 v/v).

**Results:**

In the medium containing 0.02 v/v magnetite nanoparticles, KLa was determined to be 1.89 time higher than that in magnetite nanoparticle-free broth. An improved 2.25 time higher erythromycin titer was obtained in presence of 0.02 v/v nanoparticles.

**Conclusions:**

Our results, demonstrate the potential of magnetite nanoparticles for enhancing the productivity of aerobic pharmaceutical bioprocesses.

## Background

Oxygen is one of the most important substrate influencing productivity of aerobic bioprocesses [1]. While oxygen has a low solubility in most fermentation media, the uptake of the major amount of oxygen by microorganisms during the fermentation decreases the dissolved oxygen level in liquid to less than the critical concentration, rendering oxygen the limiting factor for productivity. A significantly low rate of oxygen transfer from gas into liquid, will lead to a decreased microbial metabolic rate, thereby low fermentation performance. Therefore an adequate supply of O_2_ is required for achieving a high fermentation productivity, particularly in high-cell density bioprocesses [2] such as erythromycin fermentation by *Saccharopolyspora erythraea*.

The Oxygen Transfer Rate (OTR) is usually determined by volumetric mass transfer coefficient (KLa). This variable is affected by several factors, including composition of medium, and geometrical and operating characteristic of the bioreactor [3]. Several methods have been proposed for improving KLa, including use of more effective agitation and aeration systems, enriching air with pure oxygen, reducing gas bubbles’ size and enhancing gas hold up [2], and modifying physical properties of the medium by adding dispersed phases containing particles in size of μm [4] capable of solubilizing O_2_ more than water.

Recent studies have shown that nanomaterials have potential to positively influence the variables affecting biochemical processes. For instance, Olle et al. [4] showed that O_2_ mass transfer improves in the presence of colloidal nanoparticle dispersion. In addition, Nagi et al. [5] reported an enhanced oxygen mass transfer rate in the presence of nano-size particles.

Following this growing line of research, in the present study, first aqueous solution of magnetite nanoparticles (MNPs) was prepared. The solution was then added to the fermentation media of *S. erythraea* to examine the possible effects of magnetite nanoparticles on O_2_ transfer rate and final titer of fermentation product.

## Methods

### Materials

The erythromycin-producing strain *Saccharopolyspora erythraea* PTCC 1685 was obtained from Persian Type Culture Collection I-124, Iran. Soybean flour was supplied from Maxsoy Co., Iran. Chemical reagents and media were purchased from Merck or Sigma.

### Synthesis of nanoparticles

Several procedures have been developed for synthesis of iron oxide nanoparticles [6,7]. In this study, co-precipitation technique was used for synthesis of magnetite nanofluid, which is based on the simultaneous precipitation of Fe_3+_, Fe_2+_ ions in basic aqueous media [6]. Some advantages of this method include being straightforward, cheap, and environment-friendly, and producing a uniform size distribution of nanoparticles.

To prepare solution of MNPs, 23.5 g FeCl_3_.6H_2_O and 8.6 g FeCl_2_.4H_2_O were dissolved in 25 ml deionized water in an Erlenmeyer flask under Nitrogen sparging, 80°C and vigorous mechanical stirring. After 30 min, 45 ml of an aqueous solution of NH_4_OH was added to the mixture dropwise, after which the color of solution changes from light-brown to black, an indication of MNPs formation. The reaction was continued at 80°C under stirring and Nitrogen sparging conditions for 30 min to allow the substance completely crystalize. Afterwards the solution was cooled at room temperature [4,8-10]. The process reaction follows the following formula:

$$ FeC{l}_2+\kern0.5em 2FeC{l}_3+\kern0.5em 8N{H}_3+\kern0.5em 4{H}_2O\overset{1200\kern0.5em  rpm}{\to}\kern0.5em F{e}_3{O}_4+8N{H}_4Cl $$

Due to their strong magnetic properties, the synthetic MNPs were aggregated near the magnet. MNPs were then washed with deionized water three time sat the end of process. Next, NPs were dried in oven at 80°C overnight to be characterized by XRD and TEM analysis [4].

### Experimental determination of the volumetric mass transfer coefficient (KLa) by dynamic method

According to the Dynamic method [11] for determining mass transfer coefficient, first the concentration of the dissolved oxygen in the liquid phase in reduced by means of Nitrogen bubbling until the oxygen concentration falls to zero. Afterwards, by bubbling the air into the reaction container the concentration of dissolved oxygen is increased [11-13]. The KLa can then be calculated using the two-film theory. According to this theory, the rate of oxygen transfer from gas phase into liquid phase (at cell-free systems) is represented by:

1 $$ \frac{dc}{dt}={K}_La\left({C}^{\ast }-\kern0.5em {C}_L\right) $$

where dc/dt is the accumulation rate of the oxygen in the liquid phase, KLa represents the lumped volumetric mass transfer coefficient, C* denotes the saturate concentration of the dissolved oxygen in the broth, and C_L_ represents the dissolved oxygen concentration in the aqueous phase [14]. Integrating Equation 1 will result in Equation 2:

2 $$ {\displaystyle {\int}_{C_{L1}}^{C_{L2}}\frac{d{C}_L}{C^{\ast }-{C}_L}}={\displaystyle {\int}_{t1}^{t2}{K}_La.\kern0.5em dt} $$

With CL1 = 0 at t1 = 0, the integrated form of Equation (2) can be represented as the Equation 3:

3 $$ ln\frac{C^{*}-{C}_{L2}}{C^{*}}=-{K}_La.\kern0.5em {t}_2 $$

A plot of $ln\frac{C^{*}-{C}_L}{C^{*}}vs.\kern0.5em \mathrm{t}$ will result in a straight line with slope of -KLa [12,14].

### Media and cultural method

**Figure 1:**
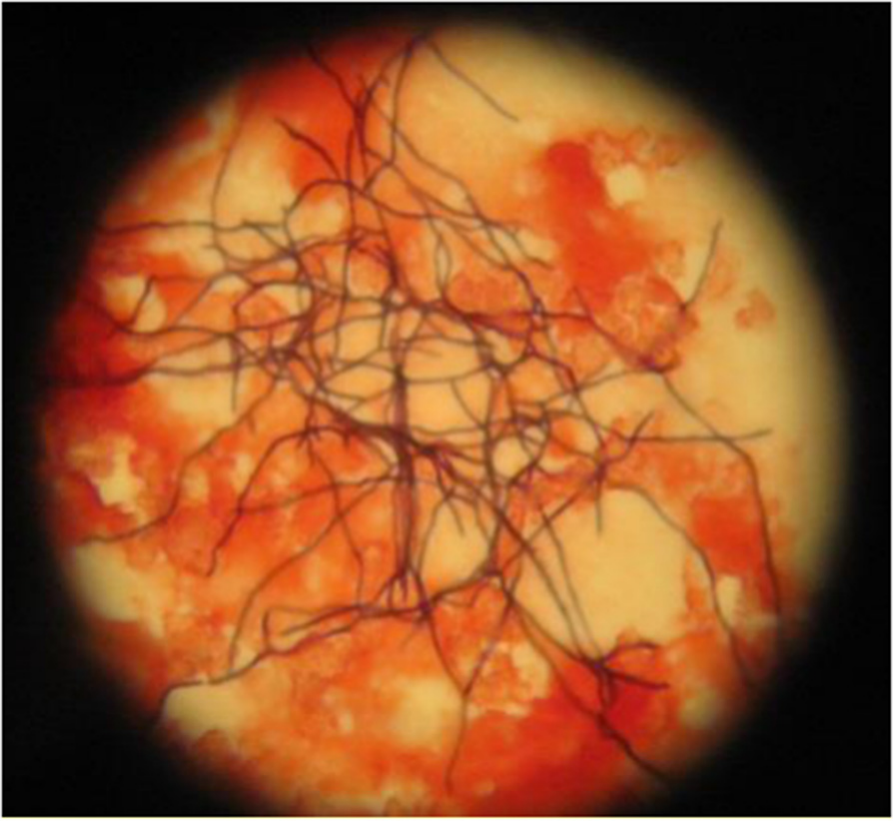
**The mycelia of strain used as inoculum.**

Spores of *S. erythraea* were produced on slants of CSL agar medium after 14 days in incubator at 30°C [15]. Ingredients of the sporulation medium used in this study was (per liter): 10 g CSL, 10 g starch, 2.5 g CaCO_3_, 3 g (NH_4_)2SO_4_, 3 g NaCl, 20 g agar, 2 ml trace element (MgSO_4_.7H_2_O, FeSO_4_.7H_2_O, ZnSO_4_.7H_2_O, CuSO_4_.5H_2_O, CoCl_2_.6H_2_O, HCL 37%), pH 7 ± 0.1 [15]. A volume of 1 ml of spore suspension of strain was inoculated in a 1000 ml Erlenmeyer flask containing 100 ml of seeding medium and incubated at 30°C and 220 rpm for 48 hours on a shaker-incubator. The composition of seeding medium used in this study was (per liter): 30 g soybean meal, 10 g glucose, 10 g glycerol, 3.5 g (NH_4_)2SO_4_, 1 g (NH_4_)2HPO_4_, 5 g CaCO_3_, pH 7 ± 0 [15]. Based on strain’s morphology (Figure 1), culture’s pH and biomass, the best inoculum was selected to be inoculated (5% v/v) into fermentation flasks. Fermentations were carried out in 9 Erlenmeyer flasks of 1000 ml, containing 150 ml of fermentation media. The composition of fermentation media was (per liter): 30 g soybean meal, 40 g dextrin, 30 g starch, 2 g(NH_4_)2SO_4_, 0.15 g (NH_4_)2HPO_4_, 10 g CaCO_3_, 50 g rapeseed oil, pH 6.8. Two concentrations of MNPs (0.005, 0.02 v/v) were added to each fermentation flask. Flasks were then put in shaker-incubator at 33°C and 220 rpm for 11 days [15-17]. All experiments were performed in triplicate in six batches. Samples of 7 ml were taken on days 6, 8, and 10 for further analysis. Investigation of *S. erythraea* morphology indicated that the hyphae of the strain were lysed on day 12, hence, no further erythromycin production was occurred.

### Erythromycin assay

Purification of erythromycin was carried out by centrifugation of the samples (4000 rpm, 20 min) followed by using magnet to separate the possibly remaining magnetite nanoparticles from the supernatant. The supernatant was then diluted with 0.2 M carbonate-bicarbonate buffer of pH9.6 and the total erythromycin was extracted with chloroform. The extracted erythromycin was then mixed with the bromophenol blue reagent. The absorbance of organic phase was measured at 415 nm by spectrophotometry [15-17].

### Statistical analysis

T-test was used to examine the significance of the mean differences. KLa was calculated from regression analysis. P < 0.05 was considered as the statistical significance.

## Results and discussion

### Nanoparticles characterization

#### NPs’ crystal structure

**Figure 2:**
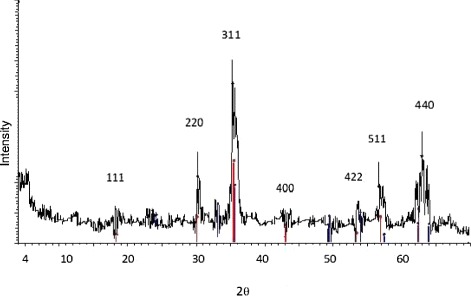
**XRD pattern of synthetic nanoparticles.**

The crystal structure of the synthetized MNPs is characterized by X-ray powder diffraction (XRD) [9]. Figure 2 displays the structural properties of the MNPs as determined by XRD (Bruker axs D4, cu, step size 0.02). The XRD pattern shows the diffraction peaks at 2θ = 18.270°, 30.035°, 35.423° (strongest line), 43.053°, 53.392°, 56.944°, 62.516°, which correspond to the (111), (220), (311), (400), (422), (511), (440) reflections, respectively [9,18-20].The XRD results show that the synthetized MNPs are well crystallized and the relative intensity of the diffraction peaks matches well with the reported XRD data for magnetite nanoparticles in the literature.

#### NPs’ size

**Figure 3:**
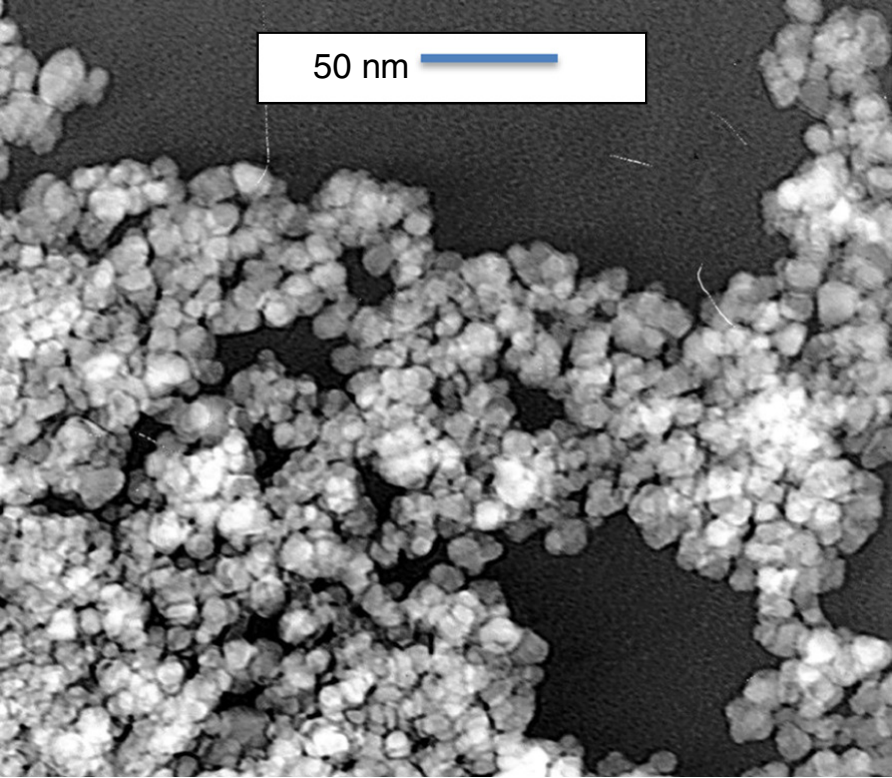
**TEM image of magnetite nanoparticles.**

Morphology and size of the prepared MNPs was determined by Transmission Electron Microscopy (TEM) (Figure 3) [10,21-23]. A small amount of dried MNPs were dissolved in ethanol. The solution was then sonicated for better dispersion. A drop of the solution was then placed on a 200 mesh Copper grid and dried in the air. The sample was analyzed by TEM at 100 KV.

**Figure 4:**
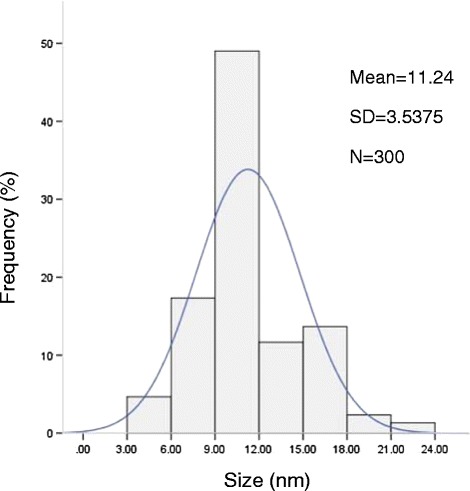
**The size distribution of magnetite nanoparticles.**

Diameter of the MNPs was determined directly from the TEM image [24]. Figure 4 displays the histogram of NPs’ size distribution (n = 300). A mean (SD) size of particles is 11.24 (3.5) nm was calculated.

### Effect of nanoparticles on mass transfer coefficient

**Figure 5:**
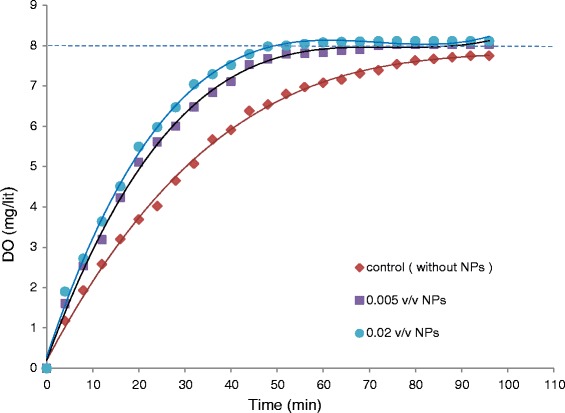
**The dissolved oxygen at different concentrations of MNPs (each value in the figure is the mean of corresponding values from 3 repeated experiments).**

**Figure 6:**
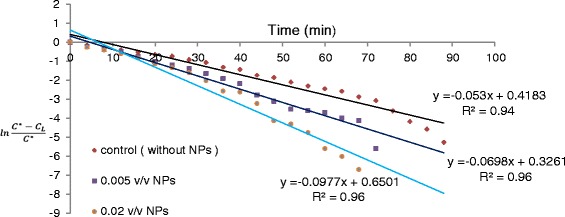
**The linearized curve of dissolved oxygen at different concentrations of MNPs (the slope of the curve represents-KLa).**

The changes in concentration of dissolved oxygen in deionized water at different concentrations of MNPs were measured during the absorption process, based on Dynamic method [11,12], and compared with nanoparticle-free water, as the control. As shown in Figure 5, at all concentrations of MNPs, the amount of DO has increased and the time for achieving saturate concentration has decreased, when compared with the control. KLa was determined using the linearized curve of DO *vs.* time (see Methods). As shown in Figure 6, MNPs positively affects the mass transfer coefficient; KLa in water containing 0.02 v/v MNPs is 1.85 times higher than that in the control.

### Effect of MNPs on erythromycin titer

**Figure 7:**
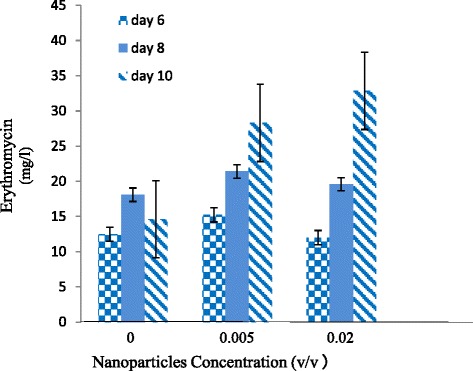
**Effect of different concentrations of nanoparticles on the production of erythromycin by S.** ***erythraea*** **(each value on the figure is the mean of corresponding values from 12 repeated experiments).**

Magnetite MNPs with initial concentrations of zero (control medium), 0.005, 0.02 v/v were used in fermentation media of *S. erythraea* and their effect on erythromycin titer was examined. As shown in Figure 7, while the maximum concentration of erythromycin is 18.1 mg/lit in the control medium, in the medium with 0.02 v/v MNPs, a maximum titer of 32.86 mg/lit has obtained which is 2.25 times higher. Erythromycin final titer (day 10) in both 0.005 and 0.02 (v/v) media shows significant difference as compared with the control (P < 0.05). Figure 6 shows that this higher titer is due to the more prolonged production of erythromycin in MNP-containing medium compared to the control medium. It could be aurged that this higher titer is due to an extended viability of the microorganism as a result of higher OTR in the presence of MNPs.

The chief objective of the present study was to explore the impact of MNPs on OTR and thereby titer of bioprocess product. MNPs are strong oxygen absorbent due to their increased surface area at nano scale. These nanoparticles have proven useful when used as recyclable oxygen carriers in aerobic fermentation [25]. A number of mechanisms proposed for the positive impact of MNPs on OTR include enhancing oxygen solubility in the fermentation medium, inducing microconvection in the surrounding fluid by Brownian motion [4], and enlarging the gas-liquid interfacial surface through being adsorbed on the air bubbles, preventing them from coalescence [26].

Results obtained in this study, which are in close agreement with the findings from previous researches [4,25-27], add weight to the notion of MNPs being candidate members of O_2_-vectors that promote the oxygen transfer in stirred aerobic bioprocesses.

Composition of fermentation medium plays an important role in the titer of secondary metabolites and the cost of fermentation product [15]. Because the erythromycin producing bacterium, *S. erythraea* is an aerobic actinomycete, proper oxygenation is crucial to achieve a high yield of this substance. Our results clearly indicate that presence of MNPs remarkably enhance the erythromycin production by *S. erythraea.*

Possible toxicity of nanoparticles is a major concern, particularly when used in pharmaceutical bioprocesses [28,29]. The fact that MNPs can be efficiently separated from fermentation medium, virtually eliminate the risk of possible toxic effects. On the other hand, the wide use of MNPs in environmental applications, including pollutant removal, toxicity mitigation, and water and waste treatment [30] indicates the safety of its use at limited doses. These advantages together with their positive impact on fermentation productivity as supported in this study, introduce use of them as a viable strategy for an improved pharmaceutical bioprocessing.

## Conclusions

The metabolic rate and growth of microbial biocatalysts are controlled by oxygen; hence, for an improved yield of aerobic bioprocesses, there is a need for strategies enabling a high rate oxygen transfer, while maintain affordable energy consumption. Our results indicate that MNPs can improve the efficiency of oxygen transfer in the fermentation medium. Use of MNPs in the erythromycin fermentation culture enhanced erythromycin titer, presumably via promotion of microbial growth and viability. The straightforward and inexpensive synthetize of these biocompatible, non-toxic and non-volatile nanoparticles, together with their positive impact on oxygen transfer rate, introduce them as promising agents for achieving an enhanced productivity of the industrial bioprocesses.
